# Barriers to, and facilitators of, education for children with disabilities worldwide: a descriptive review

**DOI:** 10.3389/fpubh.2023.1294849

**Published:** 2024-01-16

**Authors:** Kifah Bani Odeh, L. M. Lach

**Affiliations:** School of Social Work, McGill University, Montreal, QC, Canada

**Keywords:** children, disability, barriers, facilitators, education

## Abstract

**Background:**

Inclusionary ideals regarding the education of children with disabilities (CWD) are articulated in various international human rights treaties and instruments such as the United Nations Convention on the Rights (2006), the Salamanca Statement (1994), and the 2030 agenda of the UN’s Sustainable Development Goals (SDGs). In the latter, the fourth goal explicitly focuses on the removal of barriers to education and supporting access to quality, equity, and inclusion for people with disabilities. Although data regarding access to education among CWD remains scarce, it is well known that rates of their participation in education remain low, particularly among those in LMICs. The research question of this descriptive review is what are the barriers to and facilitators of education for children with disabilities worldwide aged between 6 and 18 years old?

**Methods:**

A descriptive review of literature published in English between 2013 and 2021 was conducted following the Joanna Briggs Institute (JBI) guidelines for a scoping review using the following databases: EBSCO, SocINDEX with full text (EBSCO), and ERIC (EBSCO). The search resulted in 7,072 titles and abstracts, which were narrowed down to 1,335 papers for full text review. After data extraction, 54 papers were included in the analysis, with 34 being qualitative, 10 quantitative, and 10 mixed-methods studies. The findings on the facilitators and barriers to education for children with disabilities were analyzed using the International Classification of Function, Disability and Health (ICF) and Urie Bronfenbrenner’s Ecological Framework (1979).

**Results:**

Out of the eligible studies included in our research, 40 were conducted in developing countries, while 14 studies conducted on LMICs. Of the five environmental domains in the ICF, the most significant barriers were found to be that of attitudes and services, while technology and effective communication with school staff were found to play a crucial role in facilitating the education process. Applying Bronfenbrenner’s framework, barriers occurred at the micro-system (school level), meso-system (parent and teacher communication), exo-system (services), and macro-system (education policy). Only 3 out of the 54 studies included the voices of CWD.

**Conclusion:**

Despite documented barriers, facilitators of education for CWD are underexplored, lacking research on their voices. Further investigation is needed.

## 1 Introduction

Education is a basic human right that should be available to everyone, regardless of their background (1). Accordingly, all persons are entitled to an education, regardless of gender, race, ability status, or other sources of potential discrimination (2). The United Nations (UN) has been promoting Millennium Development Goals (MDG) since 2014. One of these goals is to champion access to free and compulsory primary education for all school-age children (3). Children with disabilities (CWD) represent one of the most vulnerable groups in society in terms of their access to education, degree of community support and awareness of their rights (4, 5), but the main challenge for CWD is their lack of access to educational rights (6).

In most countries there is considerable variance in the kinds of educational opportunities that are available to disabled as compared to non-disabled children (7). Not surprisingly children in LMICs have fewer educational opportunities because of significant socio-environmental barriers (5); and CWD in LMICs s are 90% more likely to lack access to educational opportunities than CWD in developed countries (8). Further, CWD often live in fragile situations and girls, in particular, are most at risk of losing out on education (9). Also, when compared to children without disabilities, CWD face more challenges in completing all educational levels (5).

A variety of educational policies relevant to CWD have gradually evolved over time. The general trend has been to move away from the policy of streaming CWD into long-term, special education environments created to address their specific needs and, increasingly, toward Inclusive Education (IE) environments in which CWD can be integrated with non-disabled children (10). However, in order for CWD to fully participate in integrated contexts, significant adjustments must be made in school-based beliefs, rules, and procedures (11, 12). Different initiatives have been taken that facilitate education for CWD, such as supportive policies, staff training, physical infrastructure modifications, adapted assistive equipment, and the provision of emotional and economic support for the parents of CWD (13–15). Although international rights agencies typically champion both the principle of education for all and the implementation of IE policies, there remains a considerable amount of ambiguity and ambivalence regarding the translation of these policies into on-the-ground practices, especially in countries where education is under-resourced (16). Further, CWD face educational barriers as a result of difficulties associated with attempts to implement educational policies. These barriers can occur in a variety of forms: physical, cultural, social, political (e.g., policy formulation), and economic (17–20).

Additionally, parents of CWD, encounter a wide range of challenges: financial constraints, negative community attitudes toward raising CWD, and a general lack of community services and policy support for the education of CWD (21–23). In short, given the combination of lack of resources and negative attitudes, both the school environment and the community remain unfriendly toward CWD (19, 22, 24–32).

To date, studies have identified individual barriers to, and facilitators of, education for CWD. However, no systematic and comprehensive review exists that brings them all together so that policy recommendations can be made that are based on this overall understanding. The objective of this study is to conduct a comprehensive descriptive review that outlines the barriers to and facilitators of education for children with disabilities aged between 6 and 18 years old, and to highlight trends and gaps that will inform policy and future research.

In line with the Sustainable Development Goals (SDGs), it is important to consider how the findings of our study align with Goal 4 of the SDGs, which aims to ensure inclusive and equitable quality education and promote lifelong learning opportunities for all. Our study contributes to this goal by identifying the facilitators that lead to success in educating CWD and the barriers that hinder their education. By addressing these facilitators and barriers, our study provides valuable insights into how to improve education for CWD in a way that aligns with the broader global agenda of the SDGs. Additionally, conducting a descriptive scoping review allows us to identify gaps in education and provide new information about facilitators for CWD. It is important to note that our study is not limited to a specific type of disability or educational approach, making it more applicable to a broader range of contexts. This inclusivity allows our findings to be relevant and informative for various stakeholders working toward achieving Goal 4 of the SDGs.

Overall, our study contributes to the SDGs by highlighting the importance of inclusive and equitable education for all children, including those with disabilities. By understanding the facilitators and barriers in educating children with disabilities, we can work toward creating an educational environment that promotes lifelong learning opportunities and ensures quality education for every child.

## 2 Materials and methods

### 2.1 Descriptive review protocol

Given that there are no specific standards for a descriptive review, the protocol for this study adapted guidelines provided by the Joanna Briggs Institute for a scoping review. In a paper developed by Peters et al. (33), the process for conducting a descriptive review is identified: (i) define the study questions, (ii) identify relevant studies, (iii) select studies, (iv) chart the data, (v) collate the data, (vi) summarize the data, and (vii) reporting the results. The key difference between a descriptive and a scoping review is that there is no requirement for establishing inter-rater reliability at each stage of analysis.

### 2.2 Source of information

The overarching question for the descriptive review was: What are the barriers to and facilitators of education for CWD? The searches were conducted between December 2021 and January 2022. A specialist librarian helped to identify a comprehensive search strategy that combined relevant key-words. The search strategy was “barriers or challenges” AND “facilitators”: AND “Education” AND “Children” AND “Disabilities.” Search set combined the following search terms were conducted with the following databases: in Academic Search Complete (EBSCO); SocINDEX with full text (EBSCO); and ERIC (EBSCO). Data limiters were set as English abstracts in English only and 2013 as the start date. There were no geographic restrictions to studies. Results from both searches were combined and duplicates were removed. The main concepts were clarified and defined in the study as follows: the term ‘education’ includes the following contexts: special education [SE], inclusive education [IE], mainstream education, public school education, or any other type of education that targets CWD. Disability refers to a wide variety of diagnoses that reflect impairments associated with activity limitations and/or participation restrictions. Impairments may relate to movement, cognition, hearing and vision, communication, emotion, and behavior (34).

### 2.3 Eligibility criteria

The search generated 7,072 abstracts. During the initial screening of these titles and abstracts, the following questions were applied to determine which studies would be included or excluded for review at the next stage: (1) Is this a study? Yes/No/Maybe; (2) Is this about children with disabilities? (Ages 6–18)? Yes/No/Maybe; (3) Is this study about children with disabilities? Yes/No/Maybe (4) Is this about education? Yes/No/Maybe; and (5) Is this in English? Yes/No/Maybe. If the answer was “yes” or “maybe” the abstract was included in the next stage. The inclusion and exclusion criteria are listed in Table 1.

**Table 1:** The inclusion and exclusion criteria.

Inclusion criteria	Exclusion criteria
*Type of study*: observational (qualitative, quantitative, mixed)	*Type of study*: Reviews, purely descriptive or purely theoretical studies; intervention studies
*Type of participant:* The study is about children with disabilities ages 6–18 years of age; participants may be children, their caregivers, or educational stakeholders (e.g., teachers, special needs educators)	*Type of participant*: Studies of education for CWD who are less than 6 or more than 18 years of age
*Topic:* The study addresses special education (SE), inclusive education (IE) or any other context in which CWD are educated. Also included in the topic are barriers to and facilitators of the education of CWD (environmental, social, cultural, economic or in relation to any service deemed relevant to education of CWD).	*Topic*: barriers or facilitators for issues other than education
*Language*: English language only	*Language*: Published in a language other than English
*Time period:* Published between 2013 and 2021	*Time period*: Published before 2013 or after 2021

### 2.4 Selection and data charting

**Figure 1:**
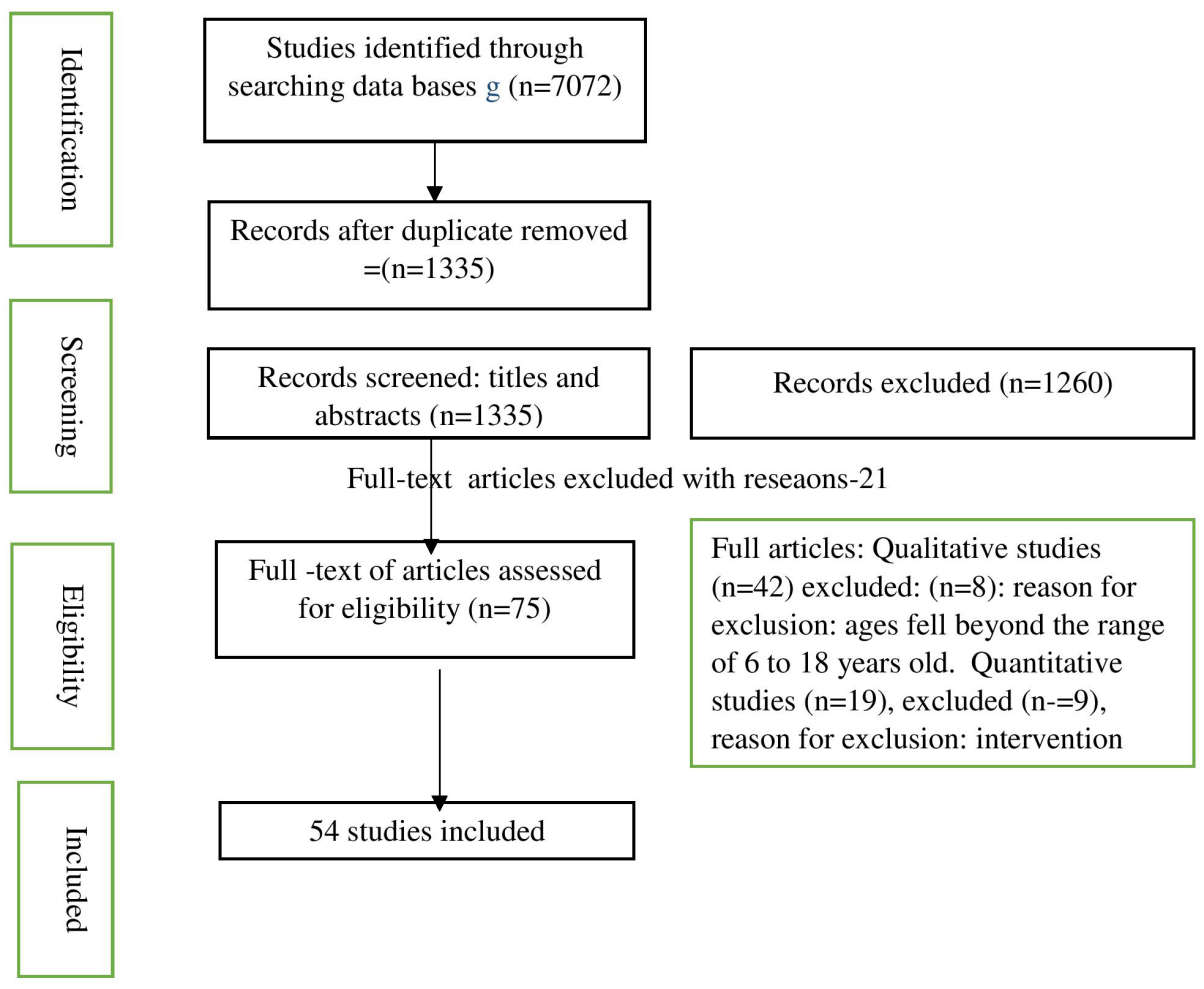
Flow chart of the search – (35).

This initial screening of titles and abstracts yielded *n* = 1,423 abstracts. After the removal of duplicates, n = 1,335 abstracts remained. At this stage, full text of each of these was reviewed to answer the following questions: (a) is this a study about CWD between 6 and 18 years of age? (b) is this a study about educational opportunities, such as Inclusive Education (IE), Special Education (SE), or any other type of education that targets CWD? (c) does this study discuss barriers to and/or facilitators of education, as reported by CWD, caregivers, and/or stakeholders in education? (d) and, is this a study using qualitative, quantitative or mixed methods design? The various stages of the search appear below in a flow chart (see Figure 1).

After applying these four criteria to the full texts of the *n* = 1,335 articles, *n* = 75 articles were accepted for a strict screening; these *n* = 75 articles consisted of *n* = 42 qualitative studies, *n* = 19 quantitative studies, and *n* = 14 mixed-method studies. Eight of the *n* = 42 qualitative studies were excluded because the ages of the children in the sample were above 18 years old. This left *n* = 34 qualitative studies. Nine of the *n* = 19 quantitative studies were excluded because they were categorized either as intervention studies (n = 6) or as experimental studies (*n* = 3). This left *n* = 10 quantitative studies. Finally, four of the n = 14 mixed-method studies were removed because (a) the research focused on special education but did not report on the educational barriers experienced by CWD (*n* = 1) and (b) the research reported on the emotional and behavioral challenges experienced by CWD at school (*n* = 3). This left *n* = 10 mixed-method studies.

The data for the study were extracted independently by the primary researcher using a data charting form. Barriers and facilitators extracted from the *n* = 54 final set of qualitative, quantitative and mixed method studies were will be approached in two steps: first, by applying the International Classification of Children and Youth (ICF) to the eligible studies selected from the research literature using the International Classification of Function, Disability, and Health, (ICF) (36) five environmental domains: (1) products and technology; (2) natural and human-made environmental change; (3) relationships and support; (4) attitudes; and (5) services, systems, and policies. Second, an additional theoretical framework, Bronfenbrenner’s ecosystems theory, will be applied the same set of studies in order to strengthen the initial analysis achieved through ICF. For example, we will compare findings on education from both high-and LMICs to see whether the types of barriers and facilitators that CWD experience differ in relation to national economy.

The World Health Organization (WHO) has developed the International Classification of Functioning, Disability and Health through many years of research and development. It is the goal of the system to provide a language and framework for describing health and health-related states across countries and settings in a consistent and comparable manner. Disability was described as an interaction between a particular health function and its contextual environment (36, 37). This model (ICF) has two main components: (i) the body domain (body functions and structures, activities and participation) and (ii) contextual factors (environment and personal factors). The environment refers to the conditions in which people live and that are external to them, such as the physical environment and the social environment. Included in the environment factors are government agencies, transportation systems, education and training, laws and regulations, as well as social attitudes that relate to these structures, services, and systems. Personal factors include characteristics that are related to one’s condition of health, such as gender, race, age, lifestyle, social background, education, occupation, and psychological characteristics (36).

The findings are presented according to which group of research participant reported them: CWD, caregivers, and stakeholders. The second way in which the findings are described use Bronfenbrenner’s ecosystems framework (38, 39). The ecological systems model founded by Bronfenbrenner will be used to synthesize the findings (40, 41). In Bronfenbrenner’s view, a child’s immediate environment, family and school, affects his or her development. Bronfenbrenner’s model also explains not only the contextual environment systems but also their interrelationships, thus uncovering a set of interlinking systems whose effects are synchronized between and among the different levels. Bronfenbrenner’s model consists of five systems: microsystem, mesosystem, exosystem, macrosystem, and chronosystem. Because these five systems are interconnected, the impact of one system upon how a child develops will affect and in turn is affected by its relationship to the others (40).

A child’s development is influenced by the level at which they are situated as well as processes of mutual influence between the individual and the environment(s), all of which should be taken into consideration when establishing the developmental trajectory (42).

Finally, the third way in which the findings are presented is, to use the findings from the 2nd approach (i.e., Bronfenbrenner ecosystems framework) to explore to what extent the barriers and facilitators reported in the literature vary based on a country’s categorization as high-income, and LMICs. Due to the global scope of this research, encompassing both high-and LMICs, it is crucial to examine the differences in barriers and facilitators between these countries and understand their distribution at various levels. This analysis will provide insights into the specific areas where barriers are most prevalent, whether at the micro or macro level, and inform targeted interventions in these countries. By identifying these disparities and understanding the interconnectedness between macro and micro levels, we can gain a comprehensive understanding of the issues surrounding education for CWD. This knowledge can guide efforts to address these obstacles effectively and promote inclusive education globally.

#### 2.4.1 Linking ICF to Bronfenbrenner ecological model

A child’s development is influenced by the level at which they are situated as well as processes of mutual influence between the individual and the environment(s), all of which should be taken into consideration when establishing the developmental trajectory (42). Using a common vocabulary and taxonomy capable of addressing developmental peculiarities and changes over time, the ICF was created to provide a multilevel approach to record aspects of children’s and adolescents’ development (36). Growth and development were fundamental factors that influenced the identification and customization of ICF material for the ICF (43). The ICF defines disability as an interaction between context-specific environmental factors and body structure and health function that can affect an individual’s execution of activities and participation in community life. The contextual factors in the ICF that let us appreciate how the environment-context affects a person’s functioning. From an ecological perspective, the focus on contextual factors that affect interventions and outcomes is also consistent. This can be attributed to the ground-breaking ecological systems theory (40) which outlines levels of social interactions that can play a role in contributing positively or negatively to child development, ranging from direct to indirect. The ecological system of childhood can be visualized as a concentric circle, in which the minor center represents the child, and each outer ring represents the system of interaction that continually surrounds it. Even though the ICF is not explicitly based on a particular theoretical framework, systems viewpoints, such as that of Bronfenbrenner, both inspired and guided its development according to the bio-ecological model (42, 44). Studies of human development are characterized by a focus on understanding the dynamic change that arises from the interaction between the developing individual and the environment in which s/he lives (43, 45). In conclusion, both the ICF and Bronfenbrenner model aim to understand the environmental context of child development; ICF provided five environmental domains to explain the factors that may act as facilitators of and barriers to meaningful activities in which persons can participate. Bronfenbrenner provided a set of nested systems, each of which is linked by several complex factors within and across the different systems, influencing their implementation and outcomes.

## 3 Results

### 3.1 Study characteristics

Characteristics of the included studies were extracted according to, the author, year of publication, study design, host country, type of disability, topic addressed (barriers, facilitators, or both), and type of participants (see Table 2).

**Table 2:** Characteristics of included studies.

Author / year of publication	Type of study design	Country	Type of disability	Educational opportunities	Research participants	Barriers/Facilitators
Alborz et al. (46)	Mixed	Iraq	CWD	IE	Education stakeholders	Barriers
Aldabas (47)	Quantitative	Saudi Arabia	Students with multiple disabilities	SE	Education stakeholders	Both
Alhuzail and Levinger (48)	Qualitative	Israel	Children with hearing loss	Education	Caregivers	Barriers
Ashbee and Guldberg (49)	Qualitative	Palestine	Autism	IE	Education stakeholders	Barriers
Bannink et al. (50)	Qualitative	Uganda	Children with neurodisabilities	IE	Combined participants.	Barriers
Bemiller (51)	Mixed	United States	Students with disabilities	IE	Education stakeholders	Both
Biggs and Hacker (52)	Qualitative	United States	Students with severe disabilities	IE	Combined participants	Both
Bouillet et al. (53)	Quantitative	Croatian countries	Students with disabilities	IE	Combined participants	Barriers
de Bruin (54)	Quantitative	Australia and United States	Students with disabilities	IE	Data review	To follow up reform in IE.
Sheehy and Budiyanto (55)	Quantitative	Indonesia	Autism	IE	Education stakeholders	Both
Buren et al. (56)	Qualitative	United States	CWD	SE	Caregivers	Both
Buren et al. (56)	Qualitative	United States	CWD	SE	Immigrants’ caregivers	Both
Comerford (57)	Qualitative	United States	Child with special need	Education	Caregiver	Barriers
Cooc and Bui (58)	Quantitative	United States	Children With Special Needs	SE	Databases-Caregivers	Barriers
Crisp et al. (59)	Qualitative	United States	Children’s use of speech-generating devices (SGD)	SE	Immigrants’ caregivers	Both
Dipeolu et al. (60)	Qualitative	United States	Children Diagnosed with Reading Disabilities	Education	Immigrant’s caregivers	Both
Fallah (61)	Mixed	United States	CWD	SE	Immigrants caregivers	Barriers
Glazzard (62)	Qualitative	England	Children with special educational needs	SE	Education stakeholders	Barriers
Goldman et al. (63)	Quantitative	United States	CWD	SE	Caregivers	Both
Graham (64)	Qualitative	Australia	Students with special educational	SE	Education stakeholders	Both
Haight et al. (65)	Qualitative	United States	learning disabilities	IE	Combined participants	Barriers
Hauwadhanasuk (66)	Qualitative	United States	Autism	SE	Immigrants’ caregivers	Barriers
Jagger and Lederer (67)	Qualitative	United States	CWD	SE	Caregivers	Barriers
Earey (68)	Qualitative	England	Dyslexia	Education	Caregivers	Barriers
Kelly and Viola (69)	Quantitative	United States	Student with disabilities	SE	CWD	Barriers
Kendall and Taylor (70)	Qualitative	United Kingdom	Children with special needs	SE	Caregivers	Barriers
Kim (71)	Qualitative	United States	Children with special needs	SE	Immigrants’ caregivers	Barriers
Lee and Park (72)	Qualitative	United States	Children with communication disorder	SE	Immigrants’ caregivers	Both
Lersilp et al. (73)	Quantitative	Chiang Mai, Thailand	Students with disabilities	SE	CWD	Both
Lim et al. (14)	Mixed	Singapore’s	Students with disabilities	IE	Education stakeholders	Barriers
Limaye (74)	Qualitative	India	CWD	IE	Education stakeholders	Barriers
Love (75)	Mixed	United States	CWD	IE	Education stakeholders	Both
Majnemer et al. (76)	Quantitative	Montreal	Children adolescents with cerebral palsy (CP)	IE	Caregivers	Barriers
Majoko (77)	Qualitative	Zimbabwe	CWD	IE	Education stakeholders	Facilitators
Makoelle (78)	Qualitative		Students with Special needs	IE	Education stakeholders	Barriers
Mann (79)	Qualitative	Australia.	intellectual impairment,	School	Caregiver	Barriers
McLeod (80)	Qualitative	United States	CWD	SE	Immigrant caregivers	Both
Mills (81)	Qualitative	Ghana	Children with Intellectual Disability	IE	Education stakeholders	Both
Mortier (82)	Qualitative	United States	Children with extensive support needs	SE	Immigrants’ caregivers	Facilitators
Mtetwa and Nyikahadzoi (83)	Mixed	Zimbabwe	CWD	Education	Caregivers	Barriers
Nahal et al. (84)	Qualitative	Palestine	Children with spina bifida	Public school	CWD	Barriers
Oliver and Singal (85)	Qualitative	England	CWD	SE	Combined participants	Both
Pretorius and Steadman (86)	Qualitative	South Africa	Child with Cerebral Palsy	Education	Caregivers	Both
Rivera et al. (87)	Mixed	United States	CWD	Collaborative teaching	Education stakeholders	Facilitators
Rossetti et al. (88)	Qualitative	United States	CWD	IE	Immigrants caregivers	Both
Schlieder et al. (89)	Qualitative	United States	Autism	IE	Combined participants	Facilitators
Sheehy and Budiyanto (55)	Mixed	Indonesia	Children with Severe Learning Disabilities	IE	Education stakeholders	Barriers
Steeley and Lukacs (90)	Qualitative	United States	CWD	SE	Immigrants caregivers	Barriers
Tanis (91)	Qualitative	United States	CWD	SE	Education stakeholders	Barriers
Thompson (92)	Qualitative	United States	CWD	SE	Immigrant caregivers	Barriers
Valeeva and Kulesza (93)	Quantitative	Poland and Russia	CWD	IE	Data review	Barriers
van der Mark and Verrest (94)	Mixed	Zimbabwe	Disabled children	School	Education stakeholders	Both
Williams (95)	Qualitative	United States	Male student in a special education	SE	Immigrants’ caregivers	Barriers
Woodley (96)	Mixed	United States	Students With Epilepsy	IE	Combined participants	Both

A majority of the studies (*n* = 34, 62.9%) were qualitative in design; *n* = 10, (18.5%) were quantitative, while (*n* = 10, 18.5%) used a mixed methods design. There was significant variability in the type of disability that studies covered. The majority were non-categorical, meaning that they included children with a range of disabilities and special needs (*n* = 38, 70.3%), while the rest (*n* = 16, 29.6%) included children with very specific diagnoses such as autism spectrum disorder, epilepsy or cerebral palsy.

Twenty of the included studies reported on both barriers and facilitators, 29 studies that reported only on barriers, and four studies reported only on facilitators. One study conducted a comprehensive analysis of trends in the development of education for children with disabilities over time (54).

When referring to educational opportunities, the studies used different terms. Most of the studies mentioned both SE and IE. Special Education was mentioned in 23 studies while Inclusive Education was mentioned in 21 studies. The rest of the studies used neither of these terms, but were still considered to be about the education of children with disabilities. The samples across these studies included caregivers (44%), education stakeholders (31%) and CWD (5.5%). 13% combined participants.

### 3.2 Using the ICF to describe environmental barriers to and facilitators of education from three different perspectives: (CWD, caregivers, and educational stakeholders)

Findings from *n* = 54 research papers were integrated and synthesized, using the ICF framework (36). Barriers and facilitators were organized into the five environmental domains that are specified in the ICF: (1) products and technology; (2) natural and human-made environmental change; (3) relationships and support; (4) attitudes; and (5) services, systems, and policies (36). The results were then presented according to which group of research participants had reported them: CWD, caregivers, and educational stakeholders. The majority of barriers were reported by parents of CWD, followed by educational stakeholders; the perspectives of CWD were sought out significantly less often. Barriers to education, as reported by different research participant groups, are summarized in Table 3.

**Table 3:** Environmental barriers to education from the perspectives of children with disabilities, caregivers, and educational stakeholders: a summary using the international classification of functioning, disability, and health (ICF).

Participant	Products and technology	Natural and built environment	Support and relationships	Attitudes	Services, systems, and policies
Children with disabilities	Not reported	School buildings and the community has not been adapted well for accessibility for CWD.	Insufficient support for CWD in schools and the ineffective interaction between school staff and CWD.	Negative attitudes and stigma toward CWD at the school level	Not reported
Caregivers	Lack of funding for parents to buy devices for CWD, poor quality of the devices, and complicated software	School buildings and the community has not been adapted.	Weak relationships between teachers and school staff, as well as poor communication strategies between parents and teachers.	Negative perception and stigma around CWD within the community and school, as well as the issue of harassment and bullying that CWD face from their peers.	Lack of financial assistance for parents to help them manage the needs of their CWD, as well as the lack of knowledge and familiarity regarding educational legislation. Challenges related to the complex bureaucratic process at school.
Educational stakeholders	Lack of knowledge of devices for CWD. lack of family support in using devices and lack of specialists in (AAC).	The physical architecture of schools posed obstacles to attending school for students with disabilities.	Lack of communication between teachers and parents at the school.	Teachers still hold negative beliefs about disability.	Insufficiently trained teachers, lack of a clear goal or plan in the Inclusive Education policy.

The first item in the ICF is products and technology, codes for Chapter 1: products and technology (5 items). For persons with disabilities, assistive devices are critical for performing daily duties and participating in social activities. These technologies, which include hearing aids, wheelchairs, Braille equipment, communication devices, and software programs, were designed to improve the quality of life of people with disabilities (36). Three studies explored barriers that were created by using technological devices: one, from the teachers’ perspective in Saudi Arabia (47), another from the parents’ perspective, in the United States (59); and one from the United States which included parents and different professionals in the field of education (52).

*Parents perspectives:* the lack of funding and specialist support has frustrated parents of children with speech-generating devices (SGD). Moreover, the quality of these devices is poor. Parents have reported difficulties with the software programs (59). *Teachers’ perspectives*: Special Education teachers in Saudi Arabia identified barriers that related to the use of augmentative and alternative communication (AAC) technology devices by children with multiple disabilities (CMD). There is a lack of knowledge about the devices among teachers, a lack of family support, a shortage of AAC specialists, and a lack of coordination between teachers and professionals in supporting AAC use in schools. Moreover, CWD often either rejected or destroyed these devices (47). *Parents and teachers’ professionals*: both parents and professionals reported on the complexity of the devices and difficulty in operating them, also, negative attitudes from peers often discourage CWD from using the AAC at school (52).

#### 3.2.1 Natural environment and human changes

The natural environment and human changes section in ICF, Chapter 2 includes 10 items. These include: the natural or physical environment, the human-change components of that environment, and the characteristics of the human populations living in that environment. The terms natural and human-made environment barriers refer to the physical accessibility of buildings and public spaces (43). In the descriptive scoping review, nine studies identified environmental barriers that were influenced by natural and constructed environment characteristics from the perspectives of CWD, caregivers, stakeholders [stakeholders (46, 50, 52, 67, 73, 74, 83, 84, 90)]. Four studies reported barriers from the parents’ perspectives (50, 67, 83, 90). Two studies cited the perspectives of CWD regarding environmental barriers (73, 84). One study was reported by different stakeholders (46), and one study interviewed parents and official school staff (52).

#### 3.2.2 CWDs perspectives

According to a study conducted in Palestine, children with spina bifida frequently have difficulty accessing buildings and streets since there are few disability-friendly environments and facilities. Due to their inability to attend school independently, they find it difficult to visit age-appropriate recreational venues, such as football games and swimming pools (84). Similar studies in Chiang Mai, Thailand found that CWD complained about stairwells, slopes, classroom doors, stairs in front of classroom doors, and elevators in the school. Furthermore, they were concerned about accessibility to activities in the school (73).

#### 3.2.3 Parents’ perspectives

for the families of CWD, the following barriers hindered access to the school: narrow doors, limited parking, broken elevators, inaccessible bathrooms, and steps in front of building entrances without ramps, which all hindered mobility for children with disabilities and their parents (50, 67). In another study, parents reported on other physical barriers. For example, there was a shortage of mobility aids like wheelchairs to assist their disabled children to go to school, particularly if their schools were located far from their homes (83). Mothers with children who have Down syndrome have complained about the lack of educational accommodations available to them (90).

#### 3.2.4 Stakeholders’ perspectives

In Iraq, parents and stakeholders remarked that in the aftermath of the conflict, school facilities were devastated, causing students to relocate to different institutions; this circumstance negatively impacted CWD. While the conflict caused a hazardous environment and a lack of security for all students, it had a greater impact on school attendance for CWD (46). The physical architecture of schools posed obstacles to attending school for students with disabilities, according to parents and professionals (52).

#### 3.2.5 Support and relationships

The support and relationships section in (ICF Chapter 3) includes teachers, parents, relatives, and friends who provide physical or emotional support, nurturing, protection, help, and support to others at home, at work, in school, or at other sites where daily activities take place (36). Thirty studies highlighted barriers to education as a result of a lack of support and relationship development at school, in the family, and in the community (14, 47–50, 52, 53, 56, 57, 60, 61, 63, 65–68, 69, 72, 74, 75, 76, 78, 80, 83, 84, 88, 90–92, 95).

#### 3.2.6 CWD’s perspectives

CWD’s sense of belonging was hindered by insufficient school assistance and ineffective interaction. Students with disabilities claim that teachers failed to relate to them or understand their negative behavior and are unable to meet the educational needs of their students (69). Children with spina bifida say they are ostracized or excluded by their classmates who are afraid to talk to them. Several students expressed their desire to attend a special needs school in order to experience normalcy and make friends (84). *Parents’ perspectives*: Teachers failed to develop communication techniques that would improve communication between teachers and parents, which led to less parental involvement in education. In the parents’ words, the school excluded them as an outsider in the educational process, which led to frustration (52, 90). The lack of communication at the school level prevented parents from taking part in school events. Therefore, they were unable to stay informed about their child’s education (48, 63). Bilingual parents of CWD with less cultural competence are more likely to have communication problems. In the absence of effective communication and discourse between multilingual CWD and the school community, such barriers may isolate them from the community. CWD had difficulty connecting with peers in the classroom because of this linguistic barrier (61). Inadequate communication hinders parents’ ability to navigate special education programs, as is evidenced by the schools’ failure to provide timely information about special education guidelines. Inadequate communication from schools can keep parents of CWD from advocating effectively for their children’s educational rights (63). Parents say they were denied the right to seek education for their CWD due to the strained relationship they had with the school and the general lack of receptivity by the staff (95). In light of the lack of support from and contact with school staff, parents appeared to be passive participants or bystanders to the educational process (72, 80, 88). Parents complained about school officials’ poor communication, resulting in their children’s academic failure. Parents often told teachers about their children’s learning challenges but the teachers ignored them or delayed responding. In addition, their children’s access to special education was compromised by a lack of solid relations with school administrators (60, 68, 83). Because of the inability of the system to give parents accurate information as well as the length of time it took for their children to receive the help they needed; many parents expressed dissatisfaction with the system (66). As a result of teacher-parent relationships that are unproductive and slow, advocating for the educational rights of CWD is invariably viewed as a ‘battle’ with care providers (56, 2014; 65, 67). The lack of educational resources was exacerbated by communication barriers. It was difficult for parents in this case to report educational resources needed by CWD (57, 92). Parents of CWD also reported that the absence of teachers supporting the child-initiated activities and mentoring the progress of CWD education also constituted barriers (75). *Stakeholders’ perspectives*: Teachers reported that strained relationships between parents and teachers negatively impacted the educational outcomes for students with disability, especially those with autism. The lack of communication at the school led teachers to feel uncomfortable instructing with CWD (14, 47, 49, 78).

#### 3.2.7 Attitudes

Within the ICF, the values, norms, and beliefs component explore how individual and social values, norms, and beliefs impact attitudes and behavior. CWD commonly face harsh criticism when their parents, teachers, or classmates do not support them. The stigma, misconceptions, and discrimination associated with CWD can discourage individuals from socializing (5). Nineteen studies documented the negative attitudes toward and beliefs concerning CWD (46, 48–50, 52, 55, 60, 61, 65, 66, 64, 74, 81, 71, 83, 84, 97, 90, 94).

*CWDs perspectives*: Spina bifida children feel angry and confused about their disfigured bodies and why they are targeted. They expressed a wide range of emotions as a result of negative attitudes. As an example, they felt enraged when they were excluded from school activities and uncomfortable about their differences. Negative attitudes and stigma were most prevalent in the school setting. Their friends insulted and humiliated them because of their disability, and they recalled the hostile behavior of their friends. Additionally, they were self-conscious about their peers’ negative attitudes and tried not to associate with them (84).

*Parents’ perspectives*: In Israel, in a Bedouin community, naming schools for CWD according to them specific impairments, such as autistic schools for children with autism, is an example of the stigma associated with the education of CWD (48). Parents complained about the community’s negative view of impairment, stating that it is either viewed as a curse, as punishment for the family, or as something worthy of sympathy (48, 61, 83, 94). In response to these kinds of negative perceptions, families have expressed concern that their CWD may be exposed to violence from community members (48). As parents have noted, some teachers also have negative views toward CWD, as demonstrated by their unwillingness to adapt their teaching methods to suit their needs (66, 71, 90). Parents report that peers showed negative attitudes toward CWD at school. A CWD with reading difficulties was subjected to name-calling, harassment, and bullying by classmates who called him “dumb, ““slow,” and “stupid” (50, 60, 65). *Stakeholders’ perspectives*: Similarly, teachers still hold negative beliefs about disability (81).

One study in Indonesia reported that 17% of teachers knew teachers who believed autism resulted from breaking a taboo; 12% knew teachers who believed autism resulted from karma; 30% believed that parents of children with autism face stigma in their community; and 24% believed teachers of children with autism face stigma (55). Teachers who sought treatment for CWD were also stigmatized. Teachers report that students with CWD who communicate using sign language face stigma both inside and outside the classroom (97). According to teachers’ observations, typically, Special Education programs for CWD are viewed negatively by the community. For that reason, CWD parents usually fail to advocate for the educational rights of their children when navigating their children’s education system (64). Different types of negative and cultural beliefs shape the education of CWD at school and community. People in the community, for example, regarded CWD from a religious perspective, maintaining that since disabled children were God’s gift, they should be compassionate and empathic toward them. Others refused to recognize CWD as family members (49). School peers bullied and laughed at CWD, in addition to refusing to interact or build relationships with them (52). According to a study in India, based on the researcher’s personal experience, CWD parents face stigma in their communities. This affects parents’ attitudes toward education for CWD, especially for girls. Some parents deny their disabled daughters the right to attend school because of their belief that educating girls is economically futile (74).

#### 3.2.8 Services, systems, and policies

Regulations, conventions, or standards are policies that are established by governments or other recognized authorities at local, regional, and national levels. Twenty-seven studies reported on barriers to the education of CWD that related to policies and other educational services (14, 46, 48–50, 52, 56, 57, 58, 59, 62, 66–68, 70–72, 74–76, 78, 80, 81, 85, 88, 91, 92).

*Parents’ perspectives*: Immigrants with disabilities and their families reported difficulties balancing work and primary care obligations due to a lack of financial assistance (88, 59). Education services were difficult to navigate for immigrant parents of CWD living in the US. In searching for educational services for their CWD, parents face challenges due to a lack of knowledge and familiarity regarding educational legislation and CWD rights for immigrant families (50, 56, 66, 71, 72). There is a lack of training in signing language among parents of children who are deaf or hard of hearing (48). In the absence of regular meetings between teachers and parents, parents feel excluded from the educational process. A variety of educational approaches and complex bureaucratic systems were more likely to present these barriers in schools with complex bureaucracies (67, 80, 92). In the classroom, there are few resources, such as therapists, who could assist teachers in understanding a child’s specific health function (57, 68, 70). There were no academic accountability procedures from the MOE that measure the quality of the community-based programs that serve CWD education. There was no evaluation of the curriculum and the educational process of teaching CWD in this center, to monitor their performance (75).

*Stakeholders’ perspectives*: Lack of educational materials, insufficiently trained teachers, and the bureaucracy of the school system has all created barriers to the implementation of education programs for CWD (14, 49, 78, 81). Autism is not addressed in special policies and parents aren’t sure how to support their autistic children. The financial burden of CWD also affects parents, who find it difficult to cover the costs of CWD related expenses. Teachers felt frustrated by the ambiguity of the Inclusive Education policy; as there are no clear goals or instructions on how to include CWD in mainstream schools (49). Schools in disadvantaged areas were evaluated solely on academic achievement. Neglecting the diversity of student populations and treating teachers as failures for not providing enough support for students with special needs is a result of the discriminatory policies and practices. Discriminatory policies left teachers emotionally exhausted and unable to work (85). In addition, teachers reported a number of challenges relating to services that increase barriers to education for CWD; broken devices, long waits for devices, limited knowledge (52). Based on other studies that cited stakeholders’ and parents’ views, teachers currently working in schools tend to have outdated and limited knowledge about disability and inclusive education (46, 62). Paraprofessionals, who are included in mainstream schools, reported that there are still problems in implementing Inclusive Education in public schools. Because school principals did not fully understand the inclusive policy and teachers were overloaded with school work, paraprofessionals were often relied upon to take full responsibility in teaching CWD (14).

#### 3.2.9 Part two: synthesis of the findings relating to facilitators of education, based on the component, environmental domains International Classification of Functioning, Disability and perspectives Health (ICF): (CWD, caregivers, and stakeholders)

The facilitators of education for CWD from three perspectives: CWD, caregivers, and stakeholders. This will be based on the five ICF domains of environment. The results of the study reported on three areas domains of the ICF: products and technology, support and relationships, and systems and policies. No findings were found under the domains natural and built environment or attitudes.

#### 3.2.10 Products and technology

In four studies, products and technology were identified as facilitators (47, 52, 73, 59). One study analyzed parent perspectives (59), one analyzed CWD perspectives (73), and one analyzed teacher perspectives (47). Another study analyzed both parents and teachers (52). Parents indicated that the devices were useful and facilitated learning. User-friendly features and good voice quality were among the parameters they mentioned (59). Teachers emphasized the need for teacher training in understanding and using the software programs of these devices. Also, the cooperation of family members can encourage the use of these devices by CWD, which can enhance their educational opportunities (47, 52).

#### 3.2.11 Support and relationships

Four studies reported that school and family support are the main facilitators of education for CWD (50, 72, 88, 96). Three studies were from teachers’ and parents’ perspectives (50, 88, 96) and one from mothers’ perspectives (72). For parents of CWD, the provision of knowledge, encouragement, optimism, and hope from other family members was crucial to the child’s educational success (50). From the perspectives of teachers and parents, there is a need to work together and communicate effectively to ensure that CWD are successful (88, 96).

#### 3.2.12 Services, systems, and policies

A total of 15 studies examined the facilitators of education for CWD in relation to services and policies (14, 46, 54, 56, 58, 63, 72, 75, 81, 82, 85–88, 93, 94). Three studies were from stakeholders’ perspectives (14, 81, 85, 87). While, eight studies documented parents’ perspectives (56, 63, 72, 82, 86, 88, 94). Three studies documented the results from two data bases sources (54, 58, 93) and one study interviewed parents and stakeholders (46).

#### 3.2.13 Parents’ perspectives

Parents recommended that caregivers who lack coping mechanisms, income-generating skills, or social support be trained, since they need to spend so much time resolving difficult situations (94). As a key financial support for parents of CWD, state financial assistance will play an important role in helping caregivers meet their children’s needs (86). Systematic advocacy is essential because agencies, service providers, and local resources such as family members and other parents make it possible. A collective mobilization of parents is more effective than individual lobbying when it comes to the rights of CWD (56, 88). Parent support groups are another way to provide emotional and informational support to other parents (72). Cultural brokers are another service that has proven to be beneficial for immigrant families. This type of group educates families about the educational system, encourages them, offers services, and provides emotional support (82). Several methods were reported for supporting parents of CWD, such as enhancing communication skills to work with school staff effectively and inviting parents to attend regular school meetings (63).

#### 3.2.14 Stakeholders’ perspectives

The teachers reported that they communicated with parents of students with disabilities using social media, such as Yahoo groups, Facebook, and regular emails. Establishing communication lines will facilitate the exchange of teaching ideas and materials, so that parents can address all educational challenges related to CWD (14). Using a co-teaching model with general education students helped support CWD’s learning and engagement. As a result of this teaching approach, students with disabilities often felt like valued members of the school community, and a sense of belonging to the school was fostered (75, 87). The teachers discussed the importance of integrating social workers into school staff in order to raise public awareness of CWD, coordinate efforts between the school and families, and advocate for the rights of children with disabilities (81). Regulation and legislation supporting inclusive education; administration of infrastructure by local government, and investment in organizational expertise in the field of disability will be good supporters (46). Knowledge, skills, and self-efficacy of school staff as well as the use of communication support strategies will increase CWD’s attendance at school (52). In particular, teachers recommend hiring volunteers from immigrant communities who are multilingual and proficient in English. These volunteers will help parents communicate more effectively and efficiently with the school and have less trouble understanding school documents. Moreover, teachers recommended hiring auxiliary employees who can assist immigrant parents when they meet with educational, health, and social services specialists, as well as direct parents to all necessary services (85).

#### 3.2.15 Personal factors

It is important to highlighted those studies in this descriptive scoping review did not report about the personal factors. Only one study, conducted in Palestine for Spina Bifida, reported on the implications of body image for students with disabilities and how their body structure became a barrier for them among their peers who excluded them from their friendship circles (84). Most studies focused on environmental barriers, rather than explicitly examining the reaction between body function and structure and the environment. This may be due to the fact that the majority of studies were reported from the perspective of parents or other stakeholders, rather than from the perspective of the children with disabilities themselves (Table 4).

**Table 4:** Environmental facilitators to education from the perspectives of children with disabilities, caregivers, and educational stakeholders: a summary using the international classification of functioning, disability and Health (ICF).

Participant	Products and technology	Support and relationships	Services, systems, and policies
Children with disabilities	Assistive technology devices can support all activities for CWD	Not reported	Not reported
Caregivers	Quality of the devices.	Effective communication, good cooperation with teachers, and knowledge about the educational system are important forms of support for parents.	Training caregivers with different skills, such as income-generating training projects, to help them cope with their CWD needs.
Stakeholders: educators, administrators, policymakers, or other relevant parties within the educational context.	Provide teacher training in the use of devices for CWD.	Not reported	Social media platforms provide new opportunities to enhance communication with parents of CWD and to inform the parents of their child’s educational status. The co-teaching model enhances CWD inclusion in schools. Staffing the school with social workers will improve communication between staff and parents of CWD. The provision of financial supports for parents, systematic advocacy and cultural brokers are advised.

### 3.3 Using Bronfenbrenner’s ecological model to describe environmental barriers to and facilitators of education for CWD

Bronfenbrenner’s (41, 98) model emphasizes how view, a child’s immediate environment such as their family and school environments affect the development of that child. Bronfenbrenner’s model consists of five subsystems: microsystem, mesosystem, exosystem, macrosystem, and chronosystem. Because these five systems are nested within one another, the impact of one system upon how a child develops will affect and in turn is affected by its relationship to the others (41, 98). Most of the barriers and facilitators in this review occurred at the microsystem level. Examples of barriers included: inadequate educational facilities, shortage of well-qualified teachers, school’s negative attitudes toward CWD (teachers’ and peers’ negative attitudes toward CWD) and the absence of family support or the presence of negative attitudes among family members toward CWD (47, 48, 52, 54, 64, 70, 72, 73, 76, 78, 84–86, 55, 90). In line with the meso-system, the barriers included the absence of communication between teachers and parents (54, 63, 66, 72, 80, 85, 88, 92, 95). At the exo-system level, the adequacy of local services to support parents of CWD indirectly affected their children’s education as parents are often assumed to be the ones who are mainly responsible for overcoming barriers (53, 55, 65, 95).

At the macro level, the reviewed studies show that similar constraints occur within all schools and that these constraints are shaped by the educational policies of each nation. For instance, lack of access to training and support for teachers and administrators is a function of how school boards prioritize disability-related training. As a macrosystem, the education of CWD may have been affected by the intersection of barriers at all levels for example, the lack of clarity regarding IE policy affected micro-level adjustments to the curriculum for CWD, while the lack of state-organized services affected parents’ involvement in education at the macro-level, indirectly affecting the education of CWD (46, 50, 74).

Regarding facilitators at the micro level, support for continuous training of teachers, availability of adapted educational materials, and having a positive attitude toward education were leading facilitators involved in encouraging CWD to learn and attend school (46, 47, 52, 80, 89). From a meso-system perspective, strengthening the relationships between parents and the teachers and other school staff indirectly affected the education of CWD (53, 59, 96). From an exosystem perspective parental support from local organizations, particularly financial assistance to face economic hardship or provide assistance with CWD-specific services like assistive equipment, was a key facilitator (66, 86, 88). The state, at the macro level, can provide a different of support for CWD (Table 5). Programs that fund advocacy, provide financial support, and education policies that promote educational services and supports for CWD were identified (54, 63, 88, 93, 96).

**Table 5:** Using Bronfenbrenner’s ecological model to describe environmental barriers to and facilitators of education for CWD.

Bronfenbrenner’ Systems	Barriers	Facilitators
Microsystem	Inadequate educational facilities, a lack of assistive technology devices, and inadequate curriculum adaptations for CWD. A shortage of well-qualified teachers and inadequate training for teachers on how to deal with CWD. Negative attitudes toward CWD among teachers and peers, and a lack of support for preventing bullying and promoting inclusion.	Continuous training of teachers working with CWD and ensure the availability of educational materials. Providing high-quality and readily available assistive devices is crucial. Implementing a school peer circle friendships initiative can provide invaluable support to CWD.
Mesosystem	A lack of communication between parents and teachers, as well as a multitude of bureaucratic procedures that can hinder effective communication between teachers and parents.	Positive relationships between teachers and parents, as well as the amount of support fathers provide to their children with regard to education.
Exosystem	Unsupportive policies at the level of schools or communities. A lack of parental engagement at school and community levels. A lack of effective and well-funded teacher training programs. A lack of resources and education regulations for children with specific disabilities, such as autism.	Organizations that provide assistance for parents and parent support groups.
Macrosystem	public cultural context can have an impact on the education of CWD. Lak of national programs and resources for helping immigrant families overcome obstacles. Education policies supporting students with disabilities do not go far enough to encourage schools to tailor their curricula to meet the needs of children with disabilities.	A state’s financial support services for parents of CWD, as well as a systematic advocacy effort among parents of children with disabilities. A dedicated community training center that provides support and resources for parents of CWD. Promoting educational policies and providing teachers with relevant new skills and information to better support the needs of children with disabilities.

### 3.4 A comparison of multisystemic barriers to and facilitators of education of CWD in high-income vs. LMICs

This part of the study highlights the disparities between high and low-income countries regarding barriers to and facilitators of educational opportunities using Bronfenbrenner’s (38) ecological framework. Using the World Bank classification for categorizing high-and LMIC s, the studies included in this analysis involved *n* = 40 studies from high-income countries, while n = 14 studies were conducted in countries ranging from upper low-income to low-income.

#### 3.4.1 Characteristics of studies conducted in high and LMICs

Countries with the highest incomes were the US, England, Australia, Israel, Singapore, Saudi Arabia, Russia, Canada, Poland, and Croatia. According to the World Bank, South Africa, Indonesia, Kazakhstan, Iraq, and Thailand were categorized as upper low-income states. Uganda, Ghana, India, Zimbabwe, and Palestine were categorized as low-income countries (99). The studies relating to high-income and low-income countries are summarized below in Tables 6, 7.

**Table 6:** High income countries.

High income countries		Author citation	No. of studies
Name of country	Australia	Graham (64), Mann (79)	2
	Canada	Majnemer et al. (76)	1
	Croatian countries	Bouillet and Kudek Mirošević (53)	1
	Israel	Alhuzail and Levinger (48)	1
	Russia and Poland	Valeeva and Kulesza (93)	1
	Saudi Arabia	Aldabas (47)	1
	Singapore’s	Lim et al. (14)	1
	United Kingdom	Glazzard (62), Earey (68), Kendall and Taylor(70), Oliver, N Singal (85)	4
	United States	Bemiller(51), Biggs and Hacker (52), Buren et al. (56), Comerford (57), Cooc and Bui (58), Crisp et al. 59, Dipeolu et al. (60), Fallah et al. (61), Goldman et al. (63), Haight et al. (65), Hauwadhanasuk (66), Jagger and Lederer (67), Kelly and Viola (69), Lee and Park (72), Love (75), McLeod (80), Mortier et al. (82), Rivera et al. (87), Rossetti et al. (88), Schlieder et al. (89) Tanis (91), Thompson (92), Williams (95), Woodley (96)	27
	USA and Australia	de Bruin (54)	1
	A total of 40 studies were conducted in high income countries		

**Table 7:** Low-income countries.

Low-income countries			
Name of country	Chiang Mai, Thailand	Lersilp et al. (73)	1
	Ghana	Mills (81)	1
	India	Limaye (74)	1
	Indonesia	Sheehy and Budiyanto (55)	2
	Iraq	Alborz et al. (46)	1
	Kazakhstan	Makoelle (78)	1
	Palestine	Ashbee and Guldberg (49), Nahal et al. (84)	2
	Uganda	Bannink et al. (50)	1
	South Africa	Pretorius and Steadman (86)	1
	Zimbabwe	Majoko (77), Mtetwa and Nyikahadzoi (83), van der Mark and Verrest (94)	3
		A total of 14 studies were conducted in low-income countries	

The review analyzed a total of 21 studies from high-income countries that focused on the barriers to education for children with disabilities (CWD) (14, 48, 53, 57, 58, 61, 62, 65–71, 76, 79, 90–93, 95). Fifteen studies reported both barriers and facilitators to education (47, 51, 52, 54, 56, 59, 60, 63, 64, 72, 75, 80, 85, 88, 96). Four studies reported on the facilitators to education, of which one documented the time trend in the reform of IE education in the United States and Australia (54, 82, 87, 89).

Fifteen of the 40 studies conducted in high-income countries examined the barriers, facilitators, or both, as experienced by immigrant and indigenous parents of CWD. Thirteen of the fifteen were conducted in the United States (54, 59, 60, 61, 65, 66, 71, 72, 80, 82, 88, 92, 100). A study was conducted in England to identify the barriers experienced by parents who had relocated from Pakistan, Bulgaria, and Poland (85). Another study, was conducted in Israel, with the Bedouin populations residents living in the Negev desert in southern Israel (48). It is interesting to note that in high income countries, approximately n = 23 studies focused on barriers and/or facilitators related to access to special education (SE) rather than inclusive education (IE). Twelve studies in high-income countries addressed IE, whereas the remaining studies used the phrase “education “in general.

*High income countries barriers*: A high-income country’s barriers were identified at four systems levels (micro, meso, exo, and macro). *The barriers to education for CWD at the microsystem*: Schools were the predominant setting in which CWD and their parents’ met barriers on the path to educating their children, as reported by 15 studies. Immigrant parents of CWD, while fighting for their child’s educational rights, cited as barriers language problems, cultural issues, and a lack of awareness about the host education system. Families that were not familiar with their host language tend to be ignorant of the educational status of their CWD; also, of how SE education operates in their schools (59, 66, 71, 80, 82, 95). Due to the language barrier, CWD experienced insecurity in the classroom and social exclusion at school (90). In England, language challenges are likely to impede both the admission to and integration of CWD in school, as well as increase the possibility of discrimination, bullying, and poor self-esteem, all of which significantly impact educational outcomes for CWD (85). A study of data from a center that provides information about CWD education services in the United Statesfound that many immigrant parents of CWD seeking services came from low-income backgrounds and spoke limited English (58). This finding is consistent with the findings of (61, 85). These authors found that language barriers prevent immigrant families of CWD from enrolling their CWD in school. Cultural differences can also lead to prejudice and discrimination within the families of CWD, since a new language and culture might impede the participation and engagement of their CWD in school (61). Chinese parents in the United States have complained about cultural disparities, cultural misunderstandings, and inadequate reciprocity between them and the teachers (66). Families of CWD also report that teachers treat them harshly and that communication lacks humanity. Parents also had difficulty collaborating with the school due to bureaucracy and red tape (80). In another example, Korean immigrant mothers of CWD were treated with contempt by staff because of cultural differences. Parents from different cultures were generally treated with hostility by staff (Kim, 2,103). As a result of the range of nationalities among CWD in schools, teachers report feeling unprepared to teach such a varied ethnic and sometimes multilingual student body. Immigrant parents, on their part, expressed concern that their children did not receive appropriate attention from their teachers, and lacked faith in the school system owing to cultural differences (92). Bedouin mothers in Israel claimed that cultural differences prevented schools that taught CWD failed to understand Bedouin perceptions about disability (48).

Teachers identified another barrier to the education of CWD in classrooms, namely, the lack of adequate training (76, 91). Education for CWD has been particularly challenging owing to teaching loads and the variety of needs of CWD (14). Managing or assisting with the complicated devices used by CWD is also a difficulty for educators (47). In other instances, teachers complained of a lack of information concerning the difficulties surrounding certain sorts of disability cases, such as epileptic seizures or autistic children’s behaviors, as well as a lack of preparedness in how to handle such cases (70, 96). The negative attitudes of teachers and lack of school accommodations for CWD have negatively impacted both the motivation of CWD to attend school and their sense of belonging (52, 69, 75). Parents of CWD who are attending school for the first time typically encounter the greatest number of obstacles (63).

##### 3.4.1.1 The barriers to education for CWD at Meso-level

The lack of communication strategies between schools and parents resulted in difficult connections, which negatively impacted the parents’ perceptions of the education of CWD in general (60, 66, 80, 90). Insufficient communication between schools and parents resulted in parents being unable to obtain accurate information about their children’s educational standing as a whole; hence, parents felt alienated from the education process (68, 96). In the United States, Spanish-speaking families with CWD expressed feelings of frustration, exhaustion, and sadness as a result of poor parent-school connections. This sort of circumstance hindered the parents’ efforts to advocate for the educational rights of their CWD (88). Local members of United States military families who have CWD indicate that the lack of communication with the school over their child’s education is their major issue (67). Further, a breakdown in communication between parents and schools may have an impact on the support available to CWD in their use of assistive technology at school (47).

##### 3.4.1.2 The barriers to education for CWD at exso-system

In high-income countries, immigrants with CWD and their families report having difficulty because of the lack of available services, particularly, services relating to the language barrier and lack of information on educational schools. These are perennial concerns for all immigrant parents (72, 92, 95). Inadequate financial assistance remained a concern for immigrant parents, leading to greater difficulty in meeting the needs of their CWD (85, 88). The school’s rigid educational system, which failed to react to parents’ needs in a timely manner, is seen by parents as an impediment when seeking education for their CWD. This system consumes parents’ time and energy during the admissions process for their CWD (71, 80). Additionally, there is a paucity of local community resources available to provide educational support to CWD after school or with their schoolwork (61, 64, 66).

##### 3.4.1.3 The barriers to education for CWD at macro system

There was little planning or collaboration relating to IE, neither for teachers nor for school district funding (52, 91). There is a shortage of qualified specialists working in this field, such as speech pathologists, educational psychologists, social pedagogues, and educators; further, no inclusion policy of worth to meet their special needs (93).

##### 3.4.1.4 The barriers to school education for CWD in LMICs

**Barriers** in LMICs were classified into micro-systems and macro-systems.

##### 3.4.1.5 The barriers to education for CWD at the microsystem

A significant barrier for LMICs is the shortage of teachers’ abilities and professionalism, as well as a lack of adequate teaching and learning tools. Ghana, for example, has a hard time promoting the implementation of IE programs because there aren’t enough training and learning resources, and many of the teachers lack the pedagogical curriculum for IE (81). In Kazakhstan, the parents reported that transitioning to IE remained challenging. Teachers are not adequately prepared to teach CWD in inclusive settings. There is no adaptive curriculum that makes it possible for CWD to receive an Inclusive Education (78). Teachers in Uganda contend that challenges with IE would worsen if teachers and educational resources are not adequately supported (50). In Indonesia, there was not enough training for teachers to deal with autistic children (55). CWD still faced challenges in terms of infrastructure, educational facilities, and teaching resources in the classroom (50). There were several micro-level barriers, such as the physical inaccessibility to the school for Palestinian CWD, who were offended by their inability to engage in school recreation programs (84). Zimbabwean households were unable to pay the school tuition of their CWD or provide them with essential assistive devices because of financial restrictions (83). In a number of LMICs, school fees continue to be a serious problem, particularly for students and families that are geographically or economically disadvantaged. Families in South Africa emphasize the need of overcoming financial obstacles to aid their CWD in all aspects of life (86).

CWD were directly affected by negative attitudes both at the community and school levels. In Ghana, CWD experience societal stigma and negative attitudes from their society, which views disability as a curse of retribution against the family (81). In India as well, parents of CWD face stigma from within the community relating to disability (74). In Palestine, children with spina bifida were interviewed. They shared their experiences of negative feelings and low self-esteem connected with wheelchair use. Their physical impairment negatively impacted their psychological health (84). According to teachers in Kazakhstan, parents’ negative views toward IE hinder the academic success of their children (78).

In Indonesia, CWD are subjected to the hostile perceptions of their culture. Some teachers at school believe that autism is a consequence of breaking a taboo or of karma and is therefore a cause for embarrassment (55). Zimbabweans, on the other hand, viewed CWD with sympathy or with sorrow (83, 94).

##### 3.4.1.6 The barriers to education for CWD at macro level

One of the barriers at the macro level is the negative public perception of the disability, which has influenced the type of community support for that disability. Clans and tribes in Uganda have negative attitudes toward disability, such that, CWD are often not recognized by their father’s clan and are prevented from receiving certain family advantages, such as an inheritance. Similarly, divorced mothers with CWD are precluded from claiming financial entitlements from their former husbands (83). The same is the case in Iraq and India, where education for CWD is defined by the cultural or religious contexts (46, 74). The educational policies for CWD often seem ambiguous, both in terms of their objectives and methods (78, 81, 55). Often, the broad educational directives are neither fully understood nor implemented at the local school or district level (46). Palestinian teachers and school staff in general still have trouble understanding the difference between inclusion and integration (49). In Ghana and Indonesia, the transition to IE is difficult to implement, for teachers have difficulty carrying out policy directives on the ground. Further, the effectiveness of IE is often weakened by the ambiguity of its goals and mission (55, 81). Given the general lack of a national education policy that targets the parents of CWD and supports them with appropriate laws and government services, families living in the more remote villages and areas especially miss out (74). In short, despite the fact that IE policies were introduced as far back in Salamanca frameworks 1994, they have not been successful in fulfilling their goals at a practical level (46, 74).

##### 3.4.1.7 The facilitators to school education for CWD in high-income countries

The facilitators were documented at three levels in high-income countries: micro, meso, and macro.

##### 3.4.1.8 The facilitators to education for CWD at the microsystem

Schools play a significant role in providing support to CWD. Families in high-income countries have suggested establishing school-based initiatives for parents to advocate for their children’s educational rights. Providing information about the local country legislation, services, and regulations relating to CWD is the main recommendation of the immigrant parents of CWD to facilitate the education of CWD in host communities (88). Furthermore, parents of CWD have suggested that a professional volunteer from the school serves as a valuable resource for parents by pointing them to appropriate services and educating them about their rights. Essentially, the volunteer would serve as a bridge between the parents and the school (72). Another facilitator that was recommended by parents was to use mediators or auxiliary employees who could act as points of contact between parents and schools. Professional immigrants employed as mediators or auxiliary employees would assist with the interpretation of documents and provide assistance at meetings with educational, health, and social service professionals (82, 85). The availability of school resources, such as educational accommodations that meet the needs of CWD, is seen as a crucial component in fostering a sense of belonging among CWD (51, 76). Educating parents and teachers about the needs of CWD is crucial in facilitating their educational process (96). Technology devices serve as facilitators for CWD by assisting them in their education at school, helping them perform their schoolwork, and communicating, both in and out of school. Availability of these devices as well the establishment of partnerships between schools and the parents will encourage their use (47, 59). The co-teaching model for CWD at the school level fosters a sense of belonging. This method has not only helped CWD learn, but has also enabled them to become more socially inclusive (75). During the development of teachers’ skills, the focus was on supporting the children’s autonomy (75). A program that encourages families to help CWD integrate will increase their integration in mainstream schools. Supporting school staff will increase their self-efficacy and help them maintain a positive perception of students’ capabilities, knowledge, and skills. Other facilitators include providing clear education instruction, implementing communication support strategies, and using adaptive curriculum (52).

##### 3.4.1.9 The facilitators to education for CWD at exso-system

It was suggested to immigrant parents of CWD that providing high-quality assistance services would help them navigate the educational support system, for example, by creating, cultural brokers who would motivate all parties to act collaboratively to improve the educational rights of CWD. Services of this type would provide parents with both educational information and social support (82). The development of systemic advocacy, facilitated by the efforts and networks of local community organizations, is also fundamental in promoting CWD education (72). According to parents, parent groups and effective communication are both essential for overcoming obstacles in the education of their CWD. As a result of these types of connections, individuals are able to cooperate and advocate for themselves (88).

##### 3.4.1.10 The facilitators to education for CWD at macro system

No studies specifically explored barriers to education for CWD at high policy levels. Only two studies in high-income countries examined progress in reforming education policy for CWD: one concerning the United States and Australia, and one concerning Poland and Russia. The purpose of studies that focused on reforming education policy were two-fold: to determine the changing number of CWD attending schools over time and to identify the gaps, strengths, and weaknesses of the Inclusive Education. The studies’ findings explored the CWD educational conditions over time as a result of the reform of education policy. This could help the policy makers monitor CWD education progress (54, 93).

##### 3.4.1.11 The facilitators to school education for CWD in LMICs

In LMICs, facilitators were located at the micro-and macro-system levels. One study reported on facilitators to education (77). Five studies reported on both barriers to and facilitators of education (55, 73, 81, 86, 94).

##### 3.4.1.12 The facilitators to education for CWD at micro-system

Support for teachers through training, adequate, physical school facilities, and sufficient educational resources for CWD are considered facilitators to their education. Supportive schooling increases children’s attendance at school in South Africa (50). According to research conducted in Chiang Mai, Thailand, assistive technology (AT) has been viewed as a facilitator for CWD with all types of impairments: AT will allow children with mobility problems to access school buildings and participate in school activities whereas the white cane and reflective tape will enable pupils with vision difficulties to better navigate their surroundings. In addition, assistive communication equipment such as sophisticated electronic devices, will facilitate communication, especially for children with hearing difficulties (73). Training teachers to handle autistic children at school and teaching signing language to teachers, classmates, and families would make a significant difference in improving the academic achievement and social interactions of CWD, as well as, reduce stigma (55).

##### 3.4.1.13 The facilitators to education for CWD macro level

Parents of CWD in South Africa relied heavily on government financial assistance. Such support enables families to send CWD to educational services, such as paying school transportation fees and purchasing assistive devices for their CWD. Government assistance was a reassuring step for parents, so they could send their children to school (81). As an example, providing parents of CWD with skills that would help them minimize their poverty affects the education and training of mothers as how to care for their CWD. Combating negative attitudes in the community will help to increase acceptance of CWD (94). Also important is to invest in the development of human and organizational expertise in the field of disability, as well as to increase the education budget. Using flexible thinking in the deployment of these resources has also been viewed as a positive step in achieving IE in Iraq (46).

This part of the study concerns the disparities between high and low-income countries regarding barriers to and facilitators of educational opportunities. Special Education (SE) represented the most frequently studied educational opportunity for CWD in high-income countries. In contrast, Inclusive Education was studied mainly in LMICs. In high-income countries, the studies tended to focus on barriers to education faced by immigrant parents, such as language barriers, cultural differences, and a lack of language specific information concerning educational policy, special education services, and education legislation, with the most common being cultural differences and language. On the other hand, a common facilitator was improved communication between parents and teachers. Studies conducted in high-income countries also aimed to identify bottlenecks experienced by immigrant parents of CWD in the local educational systems. According to studies that targeted immigrant parents of CWD, the need to provide information to policymakers about the barriers to education is of high importance. One hopes that these and other research findings on this topic will influence future education policies affecting CWD, both on local and national levels. Notably, all barriers or facilitators are assessed at the micro-level of the school. In LMICs, barriers at the school level included the following: the lack of qualified teachers to teach CWD, the lack of educational accommodations for CWD, and, with respect to teachers, the ambiguity of IE policy. In terms of facilitators in high-income countries, programs that aid immigrants’ parents of CWD in navigating the educational system were mentioned. By hiring cultural brokers and employees who could mediate, the company hoped to get around language and cultural barriers. Facilitators in LMICs focused on educational accommodations for CWD that would improve their educational environments (Table 8).

**Table 8:** Summary of multisystemic barriers to and facilitators of education-Bronfenbrenner’s ecological model in high income versus low-income countries.

Type of system	High-income countries	Low-income countries	High-income countries	Low-income countries
Barriers			Facilitators	
Micro-system	Language. Cultural. Lack of awareness about the host education system. Lack of adequate training to deal with diversity of CWD in term of needs (health and education ones)	Shortage of teachers’ ability and professionalism Lack of educational resources, infrastructure adaptation Financials issues Negative attitudes (school, community and family)	Information services support CWD legislation, services, and regulations. Mediators or auxiliary. Volunteers’ employees support. Technology devices	Teacher’s training, physical school facilities Assistive technology devices.
Meso-level	lack of communication strategies between school and parents	Not reported	Not reported	Not reported
Exso-system	Lack of service to support parents	Not reported	Cultural brokers. Systemic advocacy approach. Parent support groups	Not reported
Macro-system		Negative public perceptions of the disability Ambiguity of the educational policy for CWD.	Policy IE reform Support education institutions networks	State financial support. Integrating social workers to school staff. Funding the education Coping strategies for parents of CWD

## 4 Discussion

In *n* = 19 countries around the world, n = 54 studies examined the barriers to and facilitators of education for CWD using three study designs: qualitative, quantitative, and mixed. Of the three designs, qualitative research was the most frequently used. The studies used 23 different terms to refer to disability. Educational stakeholders and caregivers were interviewed in many of the studies, but relatively few studies (*n* = 3) reported on interviews with CWD. Finally, more studies reported on barriers to education than on facilitators of education.

### 4.1 Using the ICF to describe environmental barriers to and facilitators of education from three different perspectives: (CWD, caregivers, and educational stakeholders)

Three main domains of the ICF model, namely attitudes, social support, and services and policies are most often invoked to describe barriers of, and facilitator the education of CWD. In the community and in schools, CWD and their parents continue to face stigma, discrimination, and negative beliefs and attitudes. This review also found that the lack of cooperative strategies between parents and teachers was key as this relegates parents to being a bystanders or passive participants in their child’s schooling. Lack of support for parents reduces their ability to navigate the education system, and the lack of teaching resources and clear policies reduce the teachers’ ability to meet the needs of CWD. The domains of technology products, and natural and built environments were mentioned less frequently than attitudes, social support, and services. Further, while the research has reported on barriers to school attendance for CWD, it has not included barriers that apply to CWD who have either never attended school or who have dropped out completely. Finally, it seems that the findings of existing studies focused on barriers and facilitators to education that CWD experienced within the school setting, rather than within the larger community. Additionally, barriers and facilitators impact two specific educational opportunities: Inclusive Education and Special Education.

The facilitators of education for CWD were reported far less frequently than barriers to their education, according to the International Classification of Functioning, Disability and Health (ICF) domains. Four studies addressed, facilitators related to the ICF domains of Product and Technology, Social Support, and Services (77, 82, 87, 89). The assistive devices that CWD need in the educational setting were identified as facilitators, while the provision of knowledge, encouragement, optimism, and hope from other family members was crucial to the child’s educational success from the perspectives of teachers and parents. There is a need to work together and communicate effectively to ensure that CWD are successful. Systemic advocacy is essential because agencies, service providers, and local resources such as family members and other parents make advocacy possible. A collective mobilization of parents is more effective than individual lobbying when it comes to the rights of CWD (88). In term of services, systems, and policies state financial assistance play an important role in helping caregivers meet their children’s needs, and knowledge, skills, and self-efficacy of school staff as well as the use of communication support strategies increase CWD’s attendance at school (56, 63, 66, 86). Parents and teachers, on the other hand, identified as facilitators educational resources for enhancing the academic development of CWD. However, neither parents nor teachers mentioned other kinds of activities to which CWDs also had rights and entitlements, such as leisure activities.

### 4.2 Using Bronfenbrenner’s ecological model to describe environmental barriers to and facilitators of education for CWD

At the ecological system level, several barriers and facilitators interact to hinder CWD’s access to education. Bronfenbrenner’s framework provides a comprehensive explanation of how children’s lives develop and how being a child with a disability (CWD) relates to their lives by revealing the interactions between different levels of the ecological system. In accordance with the Bronfenbrenner framework, this review shows that home environment serves as the primary setting where learning support for children occurs, while the school environment reinforces it through parental involvement. The family and school contribute to the success of CWD by creating an environment that supports their unique needs. Families can support CWD by providing access to resources and accommodations, building positive relationships, and promoting a sense of belonging. Also, the school environment plays an important role in the academic development of CWD. Bronfenbrenner’s model (39) helped synthesize findings centering the context as the child’s environment and illustrating how each layer interacts with the others to create supportive interactions that serve children well. Despite the strength of the findings in this review, limitations remain because of the scarcity of studies that deal with how the cultural and religious context of CWD might affect their education. Historically, disability has been socially constructed in different ways, e.g., as a charity, as a medical issue, as a punishment of God, Oliver and Singal (85). Bronfenbrenner’s (98) framework include values and beliefs within the cultural context, but this review shows that three studies investigated how intersectional factors such as gender, race, religion, geography or social factors interacted in ways that either promoted or impeded the education of CWD (46, 50, 74).

### 4.3 A comparison of multisystemic barriers to and facilitators of education of CWD in high-income vs. low-income countries

Concerning differences in barriers to education between LMICs and high-income countries: studies from high-income countries were mainly about immigrant parents of CWD and stressed the need to reduce language, cultural, and service barriers. LMICs, on the other hand, focused on the ambiguity of policy and the lack of educational resources. The representation of education facilitators was inadequate compared to the barriers to education, and the poor reporting made it challenging to obtain reliable information about the facilitators.

### 4.4 The implications of their findings for policymakers within the context of the global agenda for inclusive education under SDG 4.2.

Our study’s findings provide valuable insights into the facilitators and barriers to education for children with disabilities, which have significant implications for policymakers in achieving SDG 4.2. Policymakers can use these findings to guide the development of policies and interventions that promote inclusive and equitable education for all children, including those with disabilities. For example, our study identified the importance of teacher training and support as a facilitator of education for CWD. Policymakers can use this information to develop policies that prioritize teacher training and support, ensuring that teachers are equipped with the necessary skills and knowledge to provide inclusive education for children with disabilities. Additionally, our study highlighted the impact of negative cultural perceptions and theories surrounding disability on education outcomes for CWD. Policymakers can use this information to develop policies that promote positive attitudes toward disability and encourage inclusive education practices. Overall, our study’s findings can inform policymakers’ efforts to achieve SDG 4.2 by promoting inclusive and equitable education for all children. By addressing the facilitators and barriers identified in our study, policymakers can work toward ensuring that no child is left behind in accessing quality education.

### 4.5 Gaps in the research

While the perspectives of caregivers and teachers are valuable in understanding the facilitators and barriers to education for CWD, it is essential to acknowledge that the absence of the voices of CWD is inconsistent with their rights. Inclusive research practices emphasize the importance of including the voices and perspectives of individuals with disabilities in decision-making processes that directly affect them. By excluding the voices of CWD, we miss out on valuable insights and perspectives that can contribute to a more comprehensive understanding of their educational experiences. It is crucial to prioritize the participation and empowerment of CWD, ensuring that their rights to be heard and included are respected throughout the research process. In future studies, it is recommended to incorporate methods that actively involve CWD, such as participatory research approaches or inclusive data collection methods. This will help ensure that their voices are heard, their perspectives are considered, and their rights are upheld.

### 4.6 Future direction research

Future studies should shift their focus to facilitators, so that policymakers can invest in these opportunities to improve education for CWD. CWD have are being behind in education and we urgently need to develop strategies to ensure their voices are heard and that they are included in education. Let us give CWD a chance!

## Author contributions

KB: Writing – original draft. LL: Writing – original draft, Writing – review & editing.
